# What is affective technotouch (and why does it matter)?

**DOI:** 10.1080/17458927.2023.2167420

**Published:** 2023-02-14

**Authors:** Amelia DeFalco, Luna Dolezal

**Affiliations:** a School of English, University of Leeds, Leeds, UK; b Wellcome Centre for Cultures and Environments of Health, University of Exeter, Exeter, UK

**Keywords:** Touch, technology, affect, health, robots

## Abstract

This Editors’ Introduction defines the theme of ’affective technotouch’ as referring to multidimensional embodied encounters with technologies which can trigger emotional and affective responses, while also being concerned with social, political, cultural and ethical dimensions of technological touch. With reference to neuroscience and developmental studies, we outline how touch is foundational in human experience. We then discuss contemporary technologies, such as haptic gadgets and care/companion robots, which illustrate the complexities of affective technotouch. Finally, we offer critical outlines of the six contributing articles to this Special Issue on Affective Technotouch.

The past decade has seen the appearance of a range of digital jewelry, including bracelets and rings with haptic capabilities.^1^ These accessories promise users the chance to “send ‘real’ human touch over distance” (https://feelhey.com). These wearable haptic technologies, such as the *Hey Bracelet, totwoo* vibration jewelry, and *Bond Touch* bracelets, “keep love close by,” facilitating touch-based communication with loved ones. *Hey Bracelet* promises to “deliver something only loved ones can share,” by allowing users to remotely squeeze a loved one’s wrist in order to communicate longing, love and care (https://feelhey.com). Similarly, *Bond Touch* bracelets allow partners to exchange messages through vibration, promising “emotional connection” through touch (https://uk.bond-touch.com). These rudimentary communication technologies promise to “[d]efeat the distance,” a claim that has taken on special resonance over the past few years as Covid-19 has increased isolation and the resulting lack of touch has been linked to anxiety and loneliness (von Mohr, Kirsch, and Fotopoulou 2021). Digital haptic gadgets, such as digital jewelry, are not merely offering touch mediated through technology. They are facilitating a type of “affective technotouch,” an experience of touch via a technological medium which expresses or communicates an affective state, and in doing so evokes an affective response in its user.

In recent years, engineers have developed the term “affective haptics” to describe an “emerging area of research which focuses on the design of devices and systems that can elicit, enhance, or influence on [*sic*] emotional state of a human by means of sense of touch” (Tsetserukou et al. 2009). This growing field of research is concerned with the functionality of how emotions are elicited in human subjects via interactions with technology, and how emotional responses can be communicated via haptic interfaces or other technological systems. As Eid and Osman explain, affective haptics research explores “‘the acquisition of human emotions through the human touch sensory system, the processing of emotion-related haptic data to detect affect, and the display of emotional reactions via haptic interfaces. Emotions may be solely communicated through the sense of touch or coordinated/integrated with other sensory displays (such as audition or vision) in a multimedia system”’ (2016). A range of technologies fall under the remit of affective haptics, including tactile robots, artificial skins, haptic gadgets, among many others. The mechanical details of how and why touch conveys and creates a wide range of emotions are key to understanding and designing haptic technologies, but these details don’t necessarily account for how the complexities of context and the subtle operations of power determine the meanings and significance of tactile interactions between bodies and interfaces, both human and more-than-human.

This Special Issue on “Affective Technotouch” offers perspectives on intersections of touch and technology that go far beyond the functional concerns of “affective haptics,” as described by designers and engineers. We use the term “affective technotouch” to refer to multidimensional embodied encounters with technology, including, but not limited to haptic devices, like *Bond Touch* and *Hey Bracelet*, which are designed to trigger emotional and physiological responses in their users. Affective technotouch is, of course, about expressing, communicating or evoking affective responses through physical contact with a technological interface. It also describes the interaction of human bodies and technologically-inflected environments, exploring how and why those interactions produce affective responses and states. However, we use the term “affective technotouch” to also capture a concern with the social, political, cultural and ethical dimensions of technological touch, moving beyond understanding the cause-and-effect relationship between technology and a human body to look at the deeper questions and concerns which frame how these technologies are developed, implemented and experienced. Exploring affective technotouch draws attention to the human body’s emotionality, vulnerability, porousness, and its ability, as affect theory reminds us, to both affect and be affected in equal measure (see, for example: Ahmed and Stacey 2001; Anderson 2014; Classen 2012; Seigworth and Gregg 2010; Labanyi 2010; Manning 2006; Puig de la Bellacasa 2009). As a result, the topic of affective technotouch raises questions of power, vulnerability and care. As technological apparatuses come into contact with fleshy bodies, how do these material differences produce unique power differentials? How do we interpret technotouch and its affects? How is human vulnerability affected and experienced through haptic encounters with technology? What are the ethical and social consequences of affective technotouch? The essays that follow explore these questions and more. Contributions from the the fields of neuroscience, sociology, education, science and technology studies, literary studies, and philosophy, offer novel insights into the socio-political, philosophical, ethical, cultural and physiological significance of tactile encounters between humans and technology.

The centrality of touch in human experience is undeniable. Even haptic gadgets such as *Hey Bracelet* have capitalized on the insights from touch studies in their marketing materials, with the product infographics citing formative touch theorists like Tiffany Field,^2^ to support their efficacy claims. They note that touch is integral for the formation of social bonds, and, therefore, its absence in long distance relationships can have significant consequences. The *Hey Bracelet* website declares: “In all the ways we digitally communicate with each other, there’s one very important element missing: touch. That’s where the Hey bracelet comes in” (https://feelhey.com). Although the causal relationships implied in *Hey Bracelet* infographics may be questionable, the underlying claim that touch is integral for human flourishing, not to mention survival, is well established in touch studies. Indeed, it has become something of a truism to observe that human animals are made by, through, and for touch (DeFalco and Dolezal, forthcoming). Biological research demonstrates that humans, like most mammals, enter the world as relational, interdependent beings, entirely reliant on touch for survival and communication. Touch is the first sense to develop in utero and the most important sense for newborn interaction with the world. Touch is integral not only for communication and bonding, but for our early survival and health since it is the touch cells in infants’ lips that allow them to nurse (Holler 2002). In her book on the role of touch in moral philosophy, Linda Holler explores the omnipresence of touch, its role in our everyday lives, arguing that it’s “difficult to imagine life apart from the body’s tactile awareness” (2002, 15). Touch is a primary modality for negotiating identity and selfhood, for facilitating agency, and for delivering comfort and care. At the same time, it is a site of vulnerability and risk. Though humans may be born seeking contact and nourishment via touch, cultural and socio-political forces are powerful determinants of how particular bodies experience touch, mitigating or accentuating the vulnerability attendant with this most fundamental human sense.

Touch is a central experience in human care. As the development of care technologies (including care robots) increases, the opportunities for caring “technotouch” are proliferating. Touch is especially important for companion robots designed to engender emotional reactions, attachments and bonds in (and from) their users, as Mark Paterson and Margrit Shildrick explore in their contributions to this volume. Paro, for example, is a life-size animatronic harp seal pup that has been successful at calming and comforting people with dementia. Paro gives care by accepting it; users can touch, cuddle and talk to Paro, who will respond with sounds and movement. More recently, Consequential Robotics has developed MiRo, a companion robot with the appearance of a dog/rabbit hybrid, which responds to user movement and touch. Stevie is a humanoid companion robot, developed by Conor McGinn at Trinity College Dublin, that responds to social interaction, including touch, with a range of facial expressions designed to imitate and engender emotional connection. Robots like Paro, MiRo and Stevie, which are designed to engage users’ embodied affects – to touch, and be touched in the various tactile and affective meanings of the term – often provoke concern, even anxiety in academics, journalists, and activists who worry that robot care could easily exacerbate, rather than mitigate, human isolation, marginalization, even obsolescence, a perspective formulated on the assumption that so-called real social, embodied caring contact is definitively human. Touch plays an important role in such anxieties; often care robot fears are expressed in terms of contact, as the concern that robot care lacks the specialness of “human touch,” ascribing an ineffable quality to human contact (see, for example: Sharkey and Sharkey 2010, 2012; Sparrow and Sparrow 2006; Turkle 2011).

As much as touch is celebrated as the primary mechanism for infant bonding, crucial to child development and wellbeing across the lifecourse, it also involves a degree of risk. Touch is a “reversible” sense (Puig de la Bellacasa 2009, 300); we simultaneously touch and are touched by the “not-self” matter of the world. As much as skin can appear as a boundary that contains and separates the self from the world, it is, in its tactility, a reminder of human inseparability from the world. Anything I touch, touches me; I am affecting and affected (Manning 2006). Touch can be harmful, even deadly; skin is a highly permeable barrier, absorbing the world’s nourishing elements as much as its toxins (Shildrick 1997). The essays in this Special Issue explore the multiplicity and multidimensionality of affective technotouch, attentive to the power and vulnerability of touch, along with the social and ethical implications of haptic technologies. These essays consider the vulnerability of human tactility, and what happens when porous, tender, affective surfaces of organic bodies encounter synthetic matter designed for social assistance and care.

This Special Issue on “Affective Technotouch” contributes to the emerging scholarship on technology and touch through six scholarly articles that come from a wide range of disciplinary perspectives. As noted above, contributions from the the fields of neuroscience, sociology, education, science and technology studies, literary studies, philosophy, offer novel insights into the socio-political, philosophical, ethical, cultural and physiological significance of tactile encounters between humans and technology. The rest of this introductory essay gives an overview of these articles.

The opening section, “The (Bio)Mechanics of Technotouch,” includes two articles which explore the neuroscientific and physiological foundations for touch in human subjects. They give a foundation from which we can understand the functionality and significance of technotouch. India Morrison’s article, “Touching to Connect, Explore and Explain: How the Human Brain Makes Social Touch Meaningful,” explores the neuroscience behind social touch. She describes how brain systems and structures have evolved in order to give emotional meaning to touch between human subjects and the touch we use to explore physical and social worlds. Touch, as Morrison explains, is fundamental for embodied social attachment and plays a foundational role in human development, both psychologically and socially. We are primed to receive and understand touch through an affective register, and this has implications for how we understand the ethics and limitations of technotouch. This has relevance when considering how robots are being designed to mimic social touch, the central topic of Mark Paterson’s article “Social Robots and the Futures of Affective Technotouch.” Paterson explores the increasing attention paid to touch in the development of social robots (as opposed to industrial robots). He examines two recent examples of social robots, SoCoRo and HuggieBot 2.0, addressing the theoretical and interaction design implications that arise when touch is made to be a feature of human-robot interaction. Paterson explores how non-verbal communication studies can illuminate the potential for touch and motor mimicry in robot design. These considerations provide a fascinating speculation about how robots, and human interaction with them, can provide experimental spaces to explore and investigate the future of touch and touching.

The second section, “Practising Technotouch,” includes two articles which explore practices of technotouch in diverse contexts, including within dementia care and pedagogical practices within medical schools. Firstly, Margrit Shildrick’s article, “Robotic Technologies, Touch and Posthuman Embodiment in Queer Dementia Care,” investigates affective technotouch in dementia care, where the use of animal-like companion robots is becoming increasingly widespread. These robots offer companionship and physical comfort through touching and cuddling. These interactions with robots are almost as effective as contact with real animals in improving behavioral and psychological symptoms, depression, mood and quality of life. Shildrick’s analysis considers touch as both a physical event and a metaphor for emotion, where risk and vulnerability are inherent to relations between self and other, even robot-others. Considering both queer and posthuman perspectives, Shildrick’s essay considers human entanglement with technological, non-organic entities enabled through affective technotouch, raising important theoretical, ethical and practical questions regarding the use of robots in dementia care. Turning to consider pedagogical practices within medical schools, Anna Harris’s article, “Gridding Bodies: A Topographical Survey of Teaching Technotouch in Medical School,” consider how medical students learn the sensory skill of abdominal examination through interacting with “grids,” a technological interface where the flesh-and blood-patient, or parts thereof, is replicated through material infrastructures consisting of data points and geometric markings. The article draws on ethnographic field work in a “skills laboratory” in a medical school located in the Netherlands. Exploring the biopolitical implications of how bodies are mapped through gridding practices in biomedical contexts, Harris’s article explores how these grids move “off the page, off diagrams and blackboards” and “become inscribed into skin” through the practices of the students who are learning how to touch by transposing grids onto their own bodies as stand-in patients. The grids not only organize proprioception, but come to determine sensory experiences, where embodied touch-based knowledge is constituted through a complex “technoaffective engagement.”

The final section, “Imagining Technotouch,” explores speculative and creative manifestations of affective technotouch. Carey Jewitt, Ned Barker and Jürgen Steimle’s contribution, “Interactive Skin through a Social-sensory Speculative Lens,” explores Interactive Skin, epidermal devices that enhance human bodies, unsettling the boundaries between bodies and technologies. The article arises from an interdisciplinary exploratory research-collaboration with a Human-Computer Interaction (HCI) Lab and uses a speculative narrative to explore connections between the senses, society and technology. The article centers on a fictional “found archive” that includes an e-mail exchange, a research journal-note and a future advertisement, artifacts that were generated using speculative and creative methods with the HCI Lab researchers with the aim of exploring the socially-oriented and ethical potentials and pitfalls of emerging technologies. The creation and analysis of the artifacts provoked a collaborative and critical interdisciplinary engagement that had value for research and technology design, making tangible interventions in the development of Interactive Skin. The researchers also offer an engaging and creative methodology for other researchers hoping to explore the development of technologies that create opportunities for affective technotouch. Maya Caspari’s article, “Touching Imaginaries: Otherwise Worlds and Speculative Techno-touch in Wanuri Kahiu’s *Pumzi*,” explores how Afrofuturist film interrogates and extends paradigms for understanding the intersections between technology and touch. Extending work in Black studies, Caspari’s article considers how Eurocentric and liberal humanist conceptions of both touch and technology tend to be universalized as normative, even in more radical posthumanist frameworks. She uses Afrofuturist film as a point of departure to consider the relationship between race and touch, demonstrating how touch through technology is often marketed unproblematically, without any consideration of the variability of embodiment and affective interactions, nor the discrepancies in material realities in which technologies are designed, built and distributed. Through a reading of Kenyan filmmaker Wanuri Kahiu’s film *Pumzi,* which offers a techno-dystopic vision of a near-future reality, Caspari unsettles the idea of technotouch across multiple registers, while also explicating the Eurocentric biases that have shaped modernity and the normative frames through which we understand technological development and engagement.

The idea for this Special Issue emerged, in part, from the Affective Technotouch workshop co-organized by Amelia DeFalco and Luna Dolezal at the Brocher Foundation in Switzerland in June 2022. This workshop brought together a range of academics, engineers and roboticists to explore the themes of touch and technology, exploring the many practical and ethical questions that arise in relation to haptic technologies and human care. The Brocher Foundation workshop itself arose from a longer collaboration as part of the Imagining Technologies for Disability Futures project, funded by the Wellcome Trust and based at the Universities of Leeds, Sheffield, Dundee and Exeter in the UK (https://itdfproject.org). While this Special Issue includes some contributions from the Brocher Foundation workshop participants, some of the essays here come from other contributors. Overall they represent novel and developing explorations of the various forms and implications of affective technotouch.

## Note

1. The authors would like to thank Lili Golmohammadi for her thought-provoking presentation, “The commodification of touch and digital touch”, delivered at the Brocher Foundation in June 2022, which introduced us to the *Hey Bracelet* and other haptic devices marketed toward those in long distance relationships.

2. Whose work on the medical benefits of touch, particularly for preterm infants, has played a key role in the development of touch studies (Field 2014).
